# Psychometric Properties of the Self-Compassion Scale—Short Form: Study of Its Role as a Protector of Spanish Nurses Professional Quality of Life and Well-Being during the COVID-19 Pandemic

**DOI:** 10.3390/nursrep12010008

**Published:** 2022-02-08

**Authors:** Cristina Lluch-Sanz, Laura Galiana, Gabriel Vidal-Blanco, Noemí Sansó

**Affiliations:** 1 Department of Methodology for the Behavioral Sciences, University of Valencia, 46010 Valencia, Spain; cris9@alumni.uv.es; 2 Department of Nursing, University of Valencia, 46010 Valencia, Spain; Gabriel.vidal@uv.es; 3 Department of Nursing and Physiotherapy, University of the Balearic Islands, 07120 Palma, Spain; 4 Balearic Islands Health Research Institute (IDISBA), 07120 Palma, Spain

**Keywords:** self-compassion, compassion satisfaction, compassion fatigue, burnout, well-being, nurses

## Abstract

Self-compassion is a multifaceted construct that represents compassion turned inward and involves approaching one’s failure and inadequacy with kindness. To measure these self-compassionate behaviors, the Self-Compassion Scale—Short Form (SCS-SF) is one of the most widely used and has been recurrently employed in the healthcare arena. Specifically, self-compassion has been pointed out as essential for providing compassionate care and maintaining healthcare workers balance. Aim: The aim of this study is twofold: (1) to provide evidence of the psychometric properties of the SCS-SF in a sample of Spanish nurses and (2) to study of its role as a protector of Spanish nurses professional quality of life and well-being during the COVID-19 pandemic. Methods: A sample of 115 Spanish nurses was studied. Mean age was 43.79 years old (SD = 10.99); 84.3% were women. The factorial structure of the SCS-SF was studied with competitive confirmatory factor analysis (CFA). Finally, a full structural equation model was tested, in which positive and negative self-compassion predicted professional quality of life, and professional quality of life, in turn, predicted well-being. Results: Three a priori structures were compared: one-factor, two-factor, and six-factor model. The two-factor solution, positive and negative self-compassion, was retained as the best structure to represent the data. Regarding the predictive model, the two poles of self-compassion predicted professional quality of life prediction, and professional quality of life positively predicted well-being, showing a strong relationship. Conclusion: Self-compassion can be an important resource for nurses’ balance, promoting adequate professional quality of life and their well-being.

## 1. Introduction

Self-compassion, defined as the ability to hold one’s feelings of suffering with warmth, connection, and concern [1,2], is a multifaceted construct that represents compassion turned inward and involves approaching one’s own failure and inadequacy with kindness [1,2,3]. According to Neff [1,2], we can identify compassionate and uncompassionate behavior towards ourselves: self-kindness, common humanity, and mindfulness would represent compassionate behaviors, whereas their opposite poles, including self-judgement, isolation, and over-identification, would represent the uncompassionate ones [4].

To measure these self-compassionate behaviors, the Self-Compassion Scale (SCS) [2] is, by far, the most used instrument. The SCS is a 26-item self-report instrument measuring individual differences in the domains of self-compassion. A shortened version of the scale, the Self-Compassion Scale—Short Form (SCS-SF) [5], has been also developed, including only 12 items of the original SCS scale. Several structures have been hypothesized for the SCS, including one-, two-, and six-factor solutions. In its original validation, Raes et al. [5] found evidence of a six-factor structure, with a single higher-order factor of self-compassion. However, Deniz et al.’s [6] results advocated for a one-factor solution for the Turkish version of the SCS in a sample of university students. López et al. [7], in turn, found evidence of a reduced Dutch version of the SCS with 24 items, supporting a two-factor solution with one positive and one negative factor. Additionally, with the long version of the SCS, Costa et al. [8] found that the best-fitting solution was two correlated factors, positive and negative self-compassion, when compared to the six-factor solution.

Both the SCS and the SCS-SF have been widely used in the healthcare arena, as compassionate care is the core of person-centered care [9]. Specifically, self-compassion has been proven as essential for providing compassionate care and maintaining healthcare workers’ balance [10]. Indeed, self-compassion allows the healthcare professional to build resilience against stress and burnout [11] and has been associated to professional quality of life. For instance, Gustin and Wagner [12] discovered that cultivating compassion in nursing professionals improved compassion for others. Durkin et al. [13] found that more self-compassionate nurses were less likely to experience burnout. In the same way, Sansó et al. [14] found that palliative care professionals’ compassion fatigue and burnout decreased after an intervention based on mindfulness and compassion. Moreover, self-compassion has shown a moderation effect on the association between empathy and compassion fatigue in nurses [15].

Despite the vast use of the instrument, the examination of the psychometric properties of the scale in the context of healthcare professionals have been little examined and controversial. One of the first works in this area was the one developed by García-Campayo et al. [16], who found evidence of a six-factor solution in Spanish Health Service workers, both for the SCS and the SCS-SF. These authors also discovered negative relations between self-compassion and depression, anxiety, and stress and a positive relationship with mindfulness [16]. More recently, Meng et al. [17] studied the behavior of the SCS-SF in two samples of Chinese nursing students and medical workers. Whereas the results of confirmatory factor analysis (CFA) showed that the six-factor structure did not fit the data, exploratory factor analysis (EFA) supported a three-factor structure, which consisted of one positive and two negative factors [17]. Alabdulaziz et al. [18] also examined the internal structure and other psychometric properties of self-compassion in a sample of Saudi nursing students but this time using the original long-form SCS. Results confirmed, this time, a six-factor structure.

Taking into account the literature reviewed, the aim of this study is twofold: (1) to provide evidence of the psychometric properties of the SCS-SF in a sample of Spanish nurses, including the test of the structures previously found in the literature via competitive confirmatory factor analysis, and (2) to study of its role as a protector of Spanish nurses’ professional quality of life and well-being during the COVID-19 pandemic.

## 2. Materials and Methods

### 2.1. Study Design

A cross-sectional survey of Spanish PC professionals was conducted to assess self-compassion and several related variables. This cross-sectional study has been reported using the Strengthening the Reporting of Observational Studies in Epidemiology (STROBE) Statement [19].

### 2.2. Setting and Participants

The survey was conducted during March–April 2021. Professionals were accessed through the Spanish Society for palliative care (SECPAL). Participants were sampled from their lists of members and were asked to complete an online survey using SurveyMonkey, a secure and anonymous online platform that also restricts multiple survey responses. Participation was voluntary and required respondents’ informed consent.

A total of 338 palliative care professionals included in the SECPAL Directory (available on https://secpal.com/directorio-1, accessed on 1 January 2020) were contacted by email, with two reminders in lapses of three weeks. In all the invitations, professionals were asked to share and distribute the survey among their co-workers. Only nurses who currently cared for patients at the end of their lives, but not necessarily in palliative care settings, were included in this study.

A total of 278 professionals answered the survey. After removing those that did not meet the inclusion criteria and those with missing values in the main outcomes (professional quality of life), 241 remained. Out of them, 115 were nurses and therefore were included in this study.

Mean age was 43.79 years old (SD = 10.99); 84.3% were women. Most of the participants were married or living as a couple (64.3%). Details of sample characteristics can be consulted in Table 1.

Characteristics of the sample regarding gender distribution are very similar to the latest data on registered Spanish nurses, who were 84.07% women in 2020 [20].

### 2.3. Measures

To measure self-compassion, the Self-Compassion Scale—Short Form (SCS) [5], in its Spanish version [16], was used. The SCS is formed by 12 items assessing three main components of self-compassion and their opposites: self-kindness/self-judgment, common humanity/isolation, and mindfulness/over-identification. Examples of items are “Understanding of aspects I don’t like” for positive self-compassion or “Judgmental about my own flaws” for negative self-compassion. Items were scored on a 5-point Likert-type scale, from 1, “almost never,” to 5, “almost always.”

Additionally, information on gender, age, professional quality of life, and well-being was gathered. For professional quality of life measurement, the short version of the Professional Quality of Life Scale (Short-ProQOL) in its Spanish validation was used [21]. The ProQOL comprises three subscales: compassion satisfaction, compassion fatigue, and burnout [22]. Each dimension is represented in the scale by three items and scored by the use of a 5-point Likert scale (from 1, “never,” to 5, “very often”). Scores of each dimension are calculated with the sum of the three items and therefore range from 3 to 15. Compassion satisfaction can be considered low with scores of 10 or less, medium with scores between 11 and 13, and high with scores of 14 or higher; burnout, on the other hand, can be considered low with scores of 6 or less, medium with scores between 7 and 8, and high with scores of 9 or higher; and compassion fatigue can be considered low with scores of 4 or lower, medium with scores of 5, and high with scores of 6 or higher [23]. Examples of items are “I like my work as a helper” for compassion satisfaction; “I think that I might have been affected by the traumatic stress of those I help” for compassion fatigue; and “I feel trapped by my job as a helper” for burnout. Reliability estimates in this study were 0.831 for compassion satisfaction, 0.789 for compassion fatigue, and 0.848 for burnout.

For the measurement of well-being, we used the Spanish version of the Personal Wellbeing Index [24]. The scale measures personal well-being with eight items, ranging from 1 (very dissatisfied) to 5 (very satisfied). Example of items are “How satisfied are you with what you are achieving in life?” and “How satisfied are you with your personal relationships?” The scale showed adequate psychometric properties, with a reliability estimate of 0.908.

### 2.4. Data Analysis

**Figure 1:**
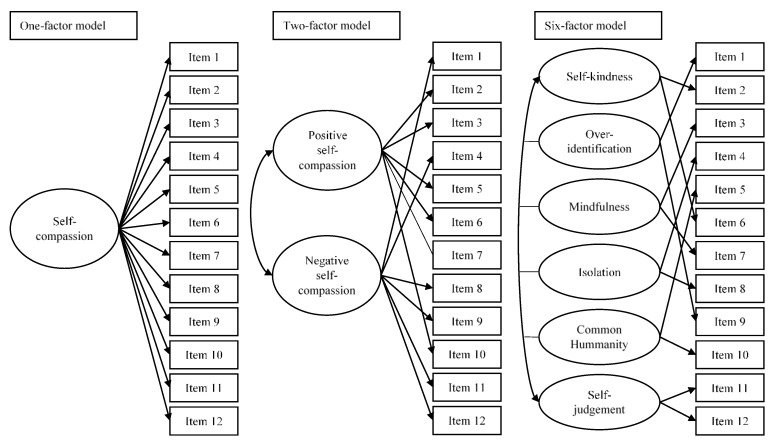
CFA models assessed for the validation of the Self-Compassion Scale—Short Form (SCS-SF).

First of all, we studied the factorial structure of the SCS-SF with competitive confirmatory factor analysis (CFA). Three CFAs were specified and tested using Mplus 8.4 [25], including (see Figure 1):

- One-factor model. This model is based on evidence provided by Deniz et al. [6], who tested a general factor of self-compassion that explained the 12 items of the SCS-SF, offering evidence that supported the unidimensional structure;
- Two-factor model. This model is based on the results of López et al. [7], Costa et al. [8], and Meng et al. [17], and hypothesized a two-correlated factor solution, including a positive self-compassionate factor and a negative one;
- Six-factor model. This model is based on the original results of Raes et al. [5], García-Campayo et al. [16], and Alabdulaziz et al. [18] and hypothesized six correlated factors, including self-kindness, common humanity, and mindfulness, which would represent compassionate behaviors, together with self-judgement, isolation, and over-identification, representing their opposite poles.

The fit of the models to the data was assessed using several goodness-of-fit indices and literature recommendations [26], including (a) the chi-square statistic; (b) the Comparative Fit Index (CFI); (c) the Root Mean Squared Error of Approximation (RMSEA); and (d) the standardized root mean squared residuals (SRMR). Following Hu and Bentler [27], a CFI of 0.950 or higher and a RMSEA or a SRMR of 0.060 or lower would indicate a very good fit of the model to the data. As the competitive CFAs were not nested, subjective criteria were used to compare the models. From this point of view, if a parsimonious model evidences adequate levels of practical fit, it is preferred over the more complex model. CFI differences (ΔCFI) of lower than 0.010 [28] or 0.050 [29] were used as cut-off criteria.

Finally, a full structural equation model (SEM) was hypothesized, specified, and tested using the best-fitting structure. In the model, positive and negative self-compassion predicted professional quality of life; and professional quality of life, in turn, predicted well-being. To assess the model fit, the same indices mentioned above were used. All models were estimated with corrected Weighted Least Squares Mean and Variance (WLSMV), the recommended procedure for ordinal and non-normal data.

## 3. Results

First of all, descriptive statistics for the variables under study, including the items of the SCS-SF and the dimensions of professional quality of life, were calculated. As displayed in Table 2, items traditionally related to negative self-compassion showed lower values than those assessing negative aspects of self-compassion. Regarding the levels of professional quality of life, nurses showed high levels of compassion satisfaction and compassion fatigue and medium levels of burnout.

For the study of the psychometric properties of the SCS-SF, three confirmatory factor analyses were specified, estimated, and assessed using the a priori structures, which are shown in Figure 1. These models were a one-factor model, a two-factor model, and a six-factor model. In Table 3, model fit indices for the three CFAs are shown.

**Figure 2:**
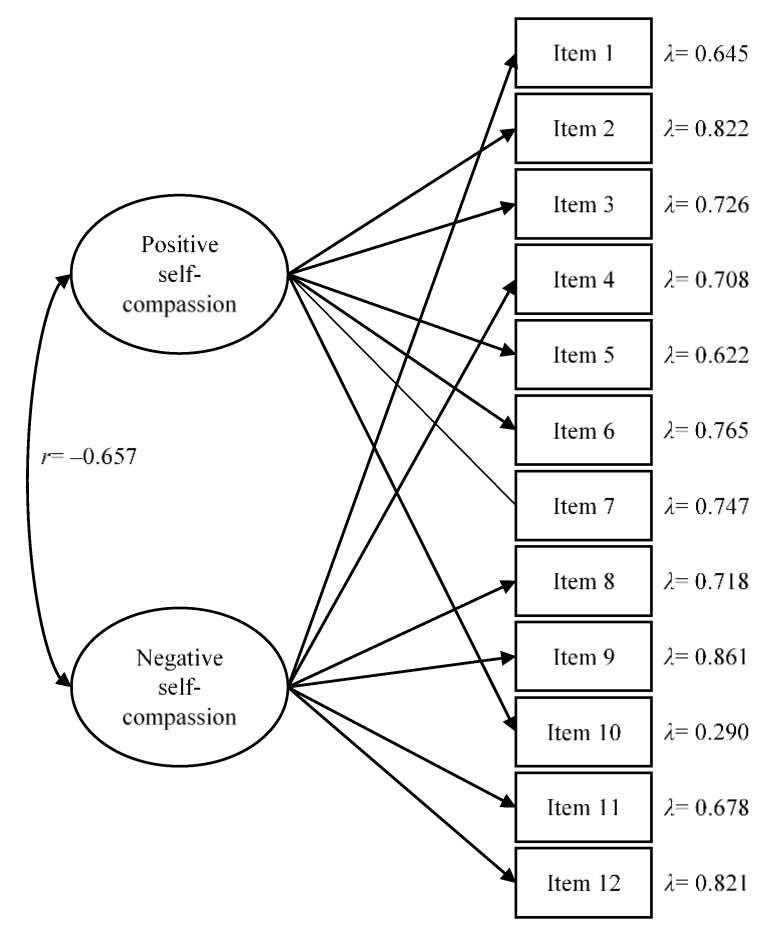
Analytical fit of the two-factor solution of the Self-Compassion Scale—Short Form (SCS-SF). For the sake of clarity, standardized values are offered. All factor loadings were statistically significant (*p* < 0.001).

When models’ fit were studied, the one-factor model was discarded, as model fit was not acceptable. The two-factor and six-factor models showed adequate fit except for the RMSEA. However, RMSEA has shown poor performance in structural models with low degrees of freedom and in small sample sizes [30]. Therefore, based on Kenny et al.’s results [30], the overall fit of these two models was considered good. When the two- and six-factor solutions were compared, CFI differences were found (Δ.027). These differences could be considered negligible attending to Little’s criteria [29]. Additionally, the correlations between self-kindness and mindfulness (*r* = 0.911) and isolation and over-identification (*r* = 0.998) in the six-factor model were extremely high, which showed no discriminant validity. Taking this into account as well as the fact that the two-factor solution was the most parsimonious one, it was retained as the best structure to represent the data. Analytical fit of the retained model can be consulted in Figure 2.

As regards reliability, estimates were adequate, with Cronbach’s alphas of 0.806 and 0.841 for positive and negative self-compassion, respectively, and omega values of 0.832 and 0.879.

With this structure, we studied the relation between self-compassion and age, gender, marital status, and main activity. Two Pearson correlations were calculated to study the relation of self-compassion with age and showed no statistically significant relationship between age and positive self-compassion (*r* = 0.129, *p* = 0.182) and a small, negative statistically significant relationship with negative self-compassion (*r* = −0.220, *p* = 0.022). Therefore, the younger the nurses were, the higher the levels of negative self-compassion. When compared by gender, statistically significant relationships were found neither for positive self-compassion (*t* (110) = 1.040, *p* = 0.301, Cohen’s d = 0.041) nor for negative self-compassion (*t* (110) = 0.417, *p* = 0.677, Cohen’s d = 0.011). The same results were found for marital status (*F* (2109) = 0.509, *p* = 0.603, *η*^2^ = 0.009; and *F* (2,109) = 1.108, *p* = 0.334, *η*^2^ = 0.020; respectively). Furthermore, main activities were analyzed, in which nurses with care and mixed activities were compared (*t* (106) = 0.174, *p* = 0.862, Cohen’s d = 0.003; and *t* (106) = 0.221, *p* = 0.826, Cohen’s d = 0.004; respectively). Descriptive statistics for these groups and for the total sample can be found in Table 4.

**Figure 3:**
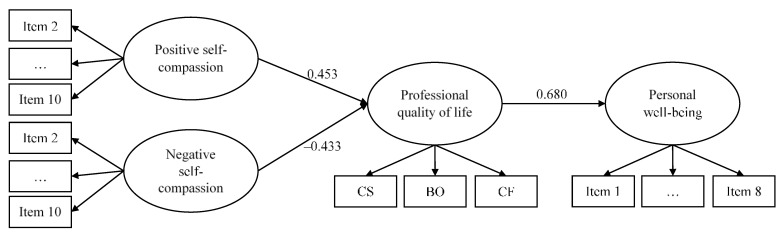
CS, compassion satisfaction; BO, burnout; CF, compassion fatigue. All the effects are statistically significant (*p* < 0.001). Factor loadings can be consulted in Table 5.

Finally, and in order to study how self-compassion could protect nurses’ professional quality of life and well-being during the COVID-19 pandemic, a structural equation model was estimated, hypothesized and tested. As displayed in Figure 3, in the model, positive and negative self-compassion predicted professional quality of life; and professional quality of life, in turn, predicted well-being.

The goodness of fit was adequate: *χ*^2^ (226) = 333.436 (*p* < 0.001), CFI = 0.907, RMSEA = 0.064 (0.049,0.079), and SRMR= 0.072. The information about the measurement part of the model is reported in Table 5, showing the factor loadings for each of the modeled factors. Factor loadings were adequate for the three latent variables: for positive self-compassion, they ranged from 0.342 (item 10) to 0.810 (item 2); for negative self-compassion, they ranged from 0.608 (item 1) to 0.857 (item 9); for professional quality of life, they ranged from 0.478 (compassion satisfaction) to −0.732 (compassion fatigue); and for wellbeing, they ranged from 0.407 (item 8) to 0.782 (item 1). For professional quality of life, compassion fatigue was indeed the most important contribution to the latent factor definition.

The main relations of the model are in the expected direction, with a high, positive, and significant coefficient from positive self-compassion to professional quality of life and a negative one from negative self-compassion to professional quality of life, with almost equal values for the two poles of self-compassion when it came to professional quality of life prediction. Finally, professional quality of life positively predicted well-being, showing a strong relationship.

## 4. Discussion

The aim of this study was to provide evidence of the psychometric properties of the SCS-SF in a sample of Spanish nurses, including the test of the structures previously found in the literature via competitive confirmatory factor analysis. Additionally, and using the best-fitting structure, this research also aimed to study the role of self-compassion as a protector of Spanish nurses’ professional quality of life and well-being during the COVID-19 pandemic. For these purposes, a sample of 115 nurses was studied.

For the first aim, three confirmatory factor analyses were specified, estimated, and assessed using three a priori structures: one-, two-, and six-factor solutions. Whereas the one-factor solution did not show an adequate fit, models of two and six factors adequately fit the data. Specifically, the two-factor solution was retained as the best-fitting model. These two factors could be caused by a method effect associated to negatively worded items, as some authors have previously found in those factors composed by only negatively formulated items [31,32,33,34]. However, these two factors could also be interpreted as two poles of self-compassion, as already defended by López et al. [7] and Costa et al. [8]. According to López et al. [7], this structure could also mean that the SCS would measure two different processes: self-compassion and self-criticism. However, the fact that the relationship of both factors has shown to be almost equal with professional quality of life leads us to the conclusion that factors could be representing, indeed, the positive and negative poles of self-compassion, supporting Neff’s recent definition of self-compassion, conceptualized as a “balance between increased compassionate and decreased uncompassionate self-responding to personal struggle” [35].

With this structure, estimates of reliability were calculated, showing appropriate values both for the positive and the negative dimensions of self-compassion. Although values were lower than the ones found by López et al. [7] and Costa et al. [8], with Cronbach’s alphas higher than 0.85 in both cases, it must be borne in mind that their studies were based on the SCS long form, and therefore, estimates could be affected by the number of items included in the dimensions. Additionally, our estimates of alpha were superior to 0.80 for both factors, indicating evidence of high reliability [36].

When self-compassion was related to demographic characteristics, no relationship with gender, marital status, or main activity was detected. This is not in line with previous literature, with results from a meta-analysis revealing that males had slightly higher levels of self-compassion than females, with a small effect size [37]. However, the subgroup of males was very small (*n* = 18), and consequently, these results may be interpreted with caution. There was found a small, statistically significant relationship between age and negative self-compassion, pointing to higher levels of negative self-compassion for the younger nurses. This is in line with results found in other populations, in which self-compassion, measured as one dimension, was found to be higher with higher age [38].

Finally, and regarding the second aim of the study, a structural equation model was hypothesized, specified, and tested. This model pointed out effects of positive and negative self-compassion on professional quality of life: higher levels of positive self-compassion predicted higher professional quality of life, whereas higher levels of negative self-compassion predicted lower levels of professional quality of life. This is in line with previous works, which supported a relationship between self-compassion and compassion fatigue and burnout [14,15]. Specifically, it is worth noting that the relationship between self-compassion and professional quality of life is very similar (indeed, it is almost equal) to the one found by Sansó et al. [39] also in Spanish nurses but before the pandemic. Therefore, self-compassion was and still is of paramount importance for preventing compassion fatigue and burnout in healthcare professionals.

This is indeed of great importance, as descriptive statistics of professional quality of life pointed to high levels of compassion satisfaction and compassion fatigue and medium levels of burnout for the sample of nurses. It is not surprising that such levels of compassion fatigue and burnout have been observed, as the COVID-19 pandemic has seriously affected healthcare professionals’ working conditions [40,41,42,43,44]. In fact, in a study on Spanish palliative care professionals surveyed just before the start of the COVID-19 pandemic, Galiana et al. [23] found medium levels of compassion fatigue. Thus, compassion fatigue levels may have increased after the pandemic, in line with recent literature [45,46,47,48,49].

In the model, we also found evidence of the predictive power of professional quality of life over personal well-being, with professionals with better professional quality of life showing higher levels of well-being. These relationships between the variables was already documented by Lizano [50], who found, in a review of human service workers, a detrimental impact of job burnout on affective, psychological, physiological, and behavioral well-being. Again, the relationship magnitude is very similar to the one found by Sansó et al. [39] in Spanish nurses before the pandemic, confirming the devastating impact that stress can have.

### Limitations

This study has some limitations, including the sample size. However, and despite the small sample size, the distribution by gender in the sample was almost exact to the one found in the data for the Spanish population. Furthermore, there is the absence of information regarding the public or private ownership of the centers or the cities in which professionals worked. Another shortcoming is the lack of test-retest reliability due to the study cross-sectional nature. Future studies in which the SCS-SF is used in bigger samples and longitudinal studies could address such limitations, offering evidence on the stability of the internal structure. Furthermore, studies in other populations, including other healthcare professionals, but also general populations would be welcomed.

## 5. Conclusions

According to our results, a two-factor solution, including the two correlated factors of positive and negative self-compassion, is the best representation of the data when using the Self-Compassion Scale—Short Form in Spanish nurses. Additionally, our results provide a better understanding of nurses’ self-compassion and its role as a protector of professional quality of life. Balanced, self-compassionate nurses, those which approach their own suffering with kindness, are better protected against burnout and compassionate fatigue and are therefore more capable of compassion satisfaction and compassionate care. Additionally, self-compassionate professionals, through this better professional quality of life, would also benefit from higher levels of well-being. Promoting self-compassion will lead to more compassionate care but also to healthier, happier professionals [51].

## Figures and Tables

**Table 1:** Sample characteristics.

Variables	Categories	*n*	%
Sex	Men	18	15.7
	Women	97	84.3
Marital status	Single	27	23.5
	Married/living as a couple	74	64.3
	Divorced	14	12.2
Main activity	Care	74	64.3
	Management	4	3.5
	Mixed	37	32.2

**Table 2:** Descriptive statistics for self-compassion in the different subgroups.

Variables	M	SD
Item 1	3.62	1.19
Item 2	3.40	1.02
Item 3	3.70	1.06
Item 4	2.50	1.32
Item 5	3.48	1.08
Item 6	2.96	1.21
Item 7	3.46	1.06
Item 8	3.05	1.19
Item 9	2.73	1.22
Item 10	2.82	1.12
Item 11	3.11	1.23
Item 12	2.70	1.12
Compassion satisfaction	13.90	1.44
Compassion fatigue	8.30	2.37
Burnout	7.30	2.20

Notes: M, mean; SD, standard deviation.

**Table 3:** Confirmatory Factor Analyses general fit.

	*χ* ^2^	df	*p*	CFI	RMSEA (90% IC)	SRMR	ΔCFI
One-factor model	261.984	54	<0.001	0.803	0.185 (0.163, 0.208)	0.094	--
Two-factor model	154.819	53	<0.001	0.903	0.131 (0.107, 0.155)	0.068	0.100
Six-factor model	113.004	39	<0.001	0.930	0.130 (0.103, 0.158)	0.055	0.027

**Table 4:** Descriptive statistics for self-compassion in the different subgroups.

Variables	Categories	Positive Self-Compassion		Negative Self-Compassion	
		M	SD	M	SD
Total sample	-	3.30	0.78	2.96	0.91
Sex	Men	3.11	0.99	2.86	1.04
	Women	3.33	0.74	2.97	0.89
Marital status	Single	3.30	0.58	3.01	0.81
	Married/living with a couple	3.34	0.79	2.88	0.96
	Divorced	3.10	1.08	3.27	0.73
Main activity	Care	3.30	0.66	2.96	0.90
	Mixed	3.27	0.99	2.92	0.97

Notes: M, mean; SD, standard deviation.

**Table 5:** Factor loadings of the SEM model.

Positive Self-Compassion	λ	Negative Self-Compassion	λ	Professional Quality of Life	λ	Personal Well-Being	λ
Item 2	0.810	Item 1	0.608	CS	0.478	Item 1	0.782
Item 3	0.714	Item 4	0.771	BO	–0.529	Item 2	0.590
Item 5	0.617	Item 8	0.678	CF	–0.732	Item 3	0.733
Item 6	0.791	Item 9	0.857			Item 4	0.657
Item 7	0.732	Item 11	0.693			Item 5	0.661
Item 10	0.342	Item 12	0.823			Item 6	0.739
						Item 7	0.676
						Item 8	0.407

Notes: CS, compassion satisfaction; BO, burnout; CF, compassion fatigue. All factor loadings resulted statistically significant (*p* < 0.001).

## Data Availability

The data that support the findings of this study are available from the corresponding author upon reasonable request.
